# Paraneoplastic Stiff Person Syndrome With Anti-amphiphysin Antibodies Presenting With Pruritus as the Initial Manifestation: An Unusual Case

**DOI:** 10.7759/cureus.35249

**Published:** 2023-02-21

**Authors:** Aiswarya Raj, Paul Alapatt, Paul Johny, Ashraf VV

**Affiliations:** 1 Department of Neurology, Aster Malabar Institute of Medical Sciences (MIMS), Kozhikode, IND

**Keywords:** breast carcinoma, anti-amphiphysin antibodies, pruritus, paraneoplastic syndrome, stiff person syndrome

## Abstract

Stiff person syndrome (SPS), also known as Stiff-man syndrome/Moersch-Woltman syndrome, is a rare disorder of the central nervous system, first described in 1956, characterized by rigidity and stimulus-triggered painful muscle spasms of predominantly axial and proximal limb muscles. There are many variants of SPS; these include the classical SPS, stiff leg syndrome, and paraneoplastic variant. The paraneoplastic variant of SPS is more common in patients with breast cancer with anti-amphiphysin antibodies, followed by colon carcinoma, lung carcinomas, thymoma, and Hodgkin’s lymphoma. A possible autoimmune origin for the disease has been proposed, including antibodies against glutamic acid decarboxylase and amphiphysin. We thus describe a case of anti-amphiphysin antibody-positive SPS, which initially manifested with generalized pruritus. After extensive investigations and removing her underlying tumor, she reported complete recovery of her symptoms.

## Introduction

Stiff person syndrome (SPS) is a rare neurological disorder with an associated autoimmune etiology. The symptoms include rigidity and stiffness as well as painful, spontaneous reflex- or action-triggered spasms in the muscles of the axial and proximal limbs. Axial rigidity with lumbar hyperlordosis and postural instability are caused by the coactivation of agonist and antagonist muscles, particularly in the paraspinal and abdominal muscles. The resulting rigidity is accompanied by potentially disabling, intermittent muscular cramps, which are often triggered by external stimuli such as jarring sounds or emotional stress [1]. High levels of antibodies (Ab) against glutamic acid decarboxylase (GAD) are associated with the predominant form of SPS, which is characterized by severe stiffness mostly affecting the spine and legs with accompanying muscle spasms [2]. A limited version of SPS (stiff limb syndrome) and a progressive variant with encephalomyelitis, stiffness, and myoclonus (progressive encephalomyelitis with rigidity and myoclonus (PERM)) are additional SPS variants that are less strongly related to GAD Ab [3]. A different variant of SPS, which we will be elaborating on in our case report, is amphiphysin Ab-associated SPS [4].

Recognition of amphiphysin antibody associated with SPS as a separate clinical syndrome has important implications, particularly concerning certain differentiating features from the typical SPS associated with GAD Ab. GAD Ab is not often paraneoplastic, although amphiphysin Ab is [5,6]. Second, intravenous immunoglobulin (IVIg) is effective against GAD Ab-associated SPS while steroids, plasmapheresis, and therapy of the underlying cancer are effective against amphiphysin Ab-associated SPS [6].

We present an unusual case of a female patient diagnosed with paraneoplastic SPS, secondary to invasive ductal breast carcinoma, who was found to be seropositive for anti-amphiphysin antibodies.

## Case presentation

A 64-year-old female patient, a known case of type 2 diabetes mellitus and systemic hypertension, presented with complaints of right lower limb pain for three months, abnormal posturing of the right foot for two months, and left lower limb pain and posturing of the left foot for two weeks. She initially had pain in her right knee associated with muscle cramps over her thigh. She also complained of stiffness in her right lower limb. She had difficulty guiding her foot into and removing her footwear probably due to stiffness. There was no history suggestive of proximal or distal muscle weakness. She also had dystonic posturing of the right foot in the form of extension of the great toe and lateral deviation of her other toes. She also developed similar complaints over her left lower limb and gradually became bedbound and she was not able to bend both knees. Gradually she developed symptoms over her right upper limb in the form of pain over her right shoulder.

There was an associated history of loss of appetite and significant weight loss over the past four months. There was also an antecedent history of generalized pruritus over a period of three months that had resolved with medication.

On examination, she had axillary lymphadenopathy and an ill-defined lump in the upper quadrant of her right breast. Neurological examination showed that higher mental functions were intact, cranial nerves were normal, and she had stiffness of both lower limbs with preserved deep tendon reflexes, flexor plantar response, complete limitations of movements at the knee, and limb resembled a log of wood. There was dystonic posturing of both feet. There were no sensory signs, no definite weakness, or bowel and bladder involvement.

**Figure 1:**
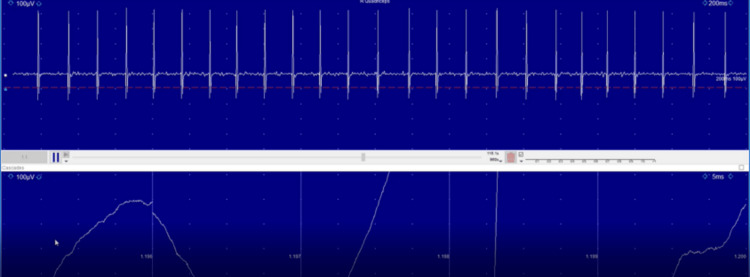
Initial electromyography study showing continuous motor activity with normal morphology

**Figure 2:**
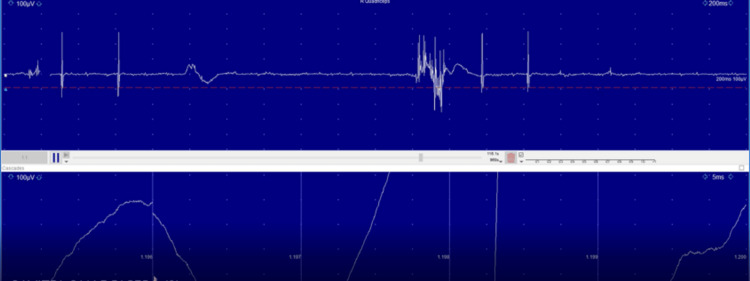
Significant decrease in activity on electromyography after administration of midazolam

Her symptoms were suggestive of a subacute onset progressive neurological disorder and a pure motor disorder. Her general examination findings were suggestive of probable breast cancer; hence, paraneoplastic neurological syndrome secondary to breast cancer was considered and SPS was the likely diagnosis based on the clinical picture. She was evaluated accordingly. Her MRI of the brain and spine were normal. An electromyography (EMG) study was carried out, which showed continuous motor activity with normal morphology, more prominent in the paraspinal muscles (Figure 1), and after administering 2 mg midazolam, the activity decreased significantly and was absent for a brief period (Figure 2). This finding was consistent with a diagnosis of SPS.

A neuronal antibody profile test was done, which was strongly positive for anti-amphiphysin antibodies. A surgical oncologist’s opinion was taken. A positron emission tomography (PET) CT was done and did not show any distant metastasis, and her TNM (tumor, node, and metastasis) staging was T2N3aM0. She underwent wide local excision with right axillary lymph node dissection and a biopsy suggestive of invasive ductal carcinoma (human epidermal growth factor receptor 2 (HER2)/neu negative, estrogen receptor (ER) positive, and progesterone receptor (PR) positive).

She was treated with muscle relaxants like diazepam, tizanidine, and baclofen and given five days of pulse steroids and later three cycles of plasma exchange therapy. She reported significant relief from pain and stiffness and is now recovering well and is currently on hormonal therapy with letrozole and maintenance steroids, undergoing neurorehabilitation, and is on follow-up with the neurology and oncology team.

## Discussion

SPS was initially reported in 1956 by Moersch and Woltman. Although its prevalence is unknown, its incidence is calculated to be one in 1,000,000 people for both sexes, even though it primarily affects women [7]. Clinical criteria such as the following are typically used to diagnose SPS: limb-specific muscular stiffness with the axial (trunk) muscles predominating in the thoracolumbar and abdominal paraspinals; absence of any other neurologic condition to explain the rigidity and stiffness; positive anti-GAD65 or amphiphysin antibodies; continuous co-contraction of both agonist and antagonist muscles; and episodes of spasms brought on by sudden noise, emotional upheaval, or tactile stimulation [8]. A paraneoplastic symptom is related with SPS in 5% of individuals. These individuals may exhibit more obvious neck and arm rigidity [8]. Manifestations in paraneoplastic SPS, however, have been shown to recede with cancer treatment [9].

Nonintrinsic membrane proteins like GAD and amphiphysin are primarily located in nerve terminals, where a pool of each protein is connected to the cytoplasmic surface of synaptic vesicles [4]. Autoantibodies against gephyrin and glycine receptors are also infrequently linked to SPS spectrum disorder [10]. Amphiphysin is not only found in gamma-aminobutyric acid (GABA)-secreting neurons, unlike GAD. The anti-glycine receptors (anti-GlyR) have a pathogenic role as they recognize extracellular epitopes of the receptor expressed in the spinal cord, brainstem, and cerebellum and glycine is a key inhibitory neurotransmitter [10]. The similar subcellular localization of GAD and amphiphysin is intriguing, as they are two known targets of CNS autoimmunity and it has been hypothesized that pathogenetic mechanisms in SPS may be linked to CNS autoimmunity directed against presynaptic components that interact with the synaptic vesicles [4]. However, all targeted antigens, including GAD and amphiphysin, are primarily cytoplasmic and have unknown pathogenicity. It is unknown if they can momentarily display an extracellular domain during neurotransmission to exert the pathogenic effect that has been hypothesized [11-13].

Pruritus has also previously been reported as an initial presentation of paraneoplastic SPS [14,15]. It is worth noting that in our patient, there was a history of pruritus, which had begun even before the onset of her reported weight loss and cachexia, and several months before the onset of her neurological manifestations. The exact mechanism for this occurrence, however, remains unknown. It has previously been postulated that the autoantibodies in SPS have a predilection for GABAergic neurons and cause a blockade of GABA synthesis [16]. This, along with the fact that GABA analogs are prescribed in the management of pruritus, might prove a clue to the underlying mechanism being linked to a relative lack of GABA in the synaptic cleft. Itching, which is frequent in non-paraneoplastic PERM as well, may also potentially be explained as a B symptom [17].

Drugs like diazepam (5-75 mg orally, four times a day) are used as part of standard SPS treatment to improve GABAergic transmission. Sodium valproate, gabapentin, baclofen, and vigabatrin can all be used to treat severe or refractory instances. Patients with GAD Ab-associated SPS can potentially benefit from IVIg treatment [18].

Amphiphysin Ab-associated SPS is an unusual presentation of a rare neuroimmunological disorder and is strongly associated with female sex, breast cancer, advanced age, EMG abnormalities, and benzodiazepine responsiveness. This condition may respond to steroids and can dramatically improve with cancer treatment [19]. Detecting anti-amphiphysin antibodies in people with severe stiffness calls for screening for breast and lung cancer.

Amphiphysin I, a 128 kD protein that is highly concentrated in nerve terminals and involved in endocytosis, has been discovered to be an autoantigen in paraneoplastic autoimmune disorders, frequently associated with breast malignancy. Although it is unclear exactly how amphiphysin antibodies contribute to the pathogenesis of paraneoplastic SPS. The postulated explanation for these paraneoplastic syndromes is that neoplastic transformation of non-neuronal tissue causes ectopic production of a neuronal protein in that tissue and ectopic expression results in neurological illness and anti-nervous system autoantibodies [20].

There are significant distinctions between amphiphysin Ab-associated and GAD Ab-associated types of SPS, even if the causes as to why they exhibit different patterns of regional involvement are yet unknown. SPS linked to GAD Ab responds to IVIg. IVIg may not be effective for amphiphysin Ab-associated SPS; however, case studies indicate that plasmapheresis, steroids, and breast cancer therapy are effective in treating the main pathophysiology [19].

Following an initial diagnosis of SPS, our patient was initially started on intravenous diazepam, baclofen, gabapentin, and tizanidine for symptomatic relief. The autoantibody panel sent was positive for anti-amphiphysin antibodies and wide local excision was carried out with right axillary lymph node dissection for her underlying breast carcinoma. She then underwent three cycles of plasma exchange therapy. In the next few days, the patient reported complete relief of pain, and in the following months, with physical rehabilitation, she reported improvement in her limb stiffness and was able to walk with support.

## Conclusions

Isolated SPS in adult female patients must be thoroughly evaluated for an underlying malignancy. In particular, a thorough physical examination must be carried out to rule out breast carcinoma. SPS which occurs as a paraneoplastic manifestation of breast carcinoma is commonly associated with anti-amphiphysin antibodies, which are recognized as a separate clinical entity, given their favorable response to steroids, plasmapheresis, and removal of the underlying tumor.
